# Bullous hemorrhagic dermatosis is an under-recognized side effect of full dose low-molecular weight heparin: a case report and review of the literature

**DOI:** 10.1186/s40164-018-0108-7

**Published:** 2018-07-06

**Authors:** Armand Russo, Susanna Curtis, Raisa Balbuena-Merle, Roxanne Wadia, Ellice Wong, Herta H. Chao

**Affiliations:** 1 Department of Internal Medicine, Yale School of Medicine and Yale Comprehensive Cancer Center, New Haven, CT 06511 USA; 2 Department of Laboratory Medicine, Yale School of Medicine, New Haven, CT 06511 USA; 3 VA Connecticut Healthcare System, Cancer Center, 950 Campbell Avenue, Mailcode 111D, West Haven, CT 06516 USA

**Keywords:** Bullous hemorrhagic dermatosis, Enoxaparin, Low molecular weight heparin

## Abstract

Bullous hemorrhagic dermatosis (BHD) is a systemic side-effect of low molecular weight heparin, characterized by multiple intra-epidermal hemorrhages distant from the site of injection. There have been several small case series and literature reviews on BHD, but none have captured a complete set of reported patients. We sought to describe a case of BHD with late diagnosis and completely summarize the existing English and Spanish literature with searches of Pubmed, Scopus, Ovid Embase and Ovid Medline. After narrowing to 33 relevant reports, we describe 90 reported cases worldwide from 2004 to 2017, in addition to a new case from our institution as a means of comparison. We found that BHD was common in elderly men (mean age 72 ± 12; male:female, 1.9:1) and typically occurred within 7 days of administration of anticoagulation (median 7 days ± 6.4) usually with enoxaparin use (66% of cases). Lesions occurred primarily on the extremities only (67.9% of cases). Coagulation testing was most often normal before administration, and the majority of patients had coagulation testing in therapeutic range during treatment. Most practitioners stopped anticoagulation if continued therapeutic intervention was no longer required (57% of cases), or changed therapy to another anticoagulation if continued treatment was required (14.3% of cases). Therapy was continued outright in 23% of patients. The lesions usually resolved within 2 weeks (mean days, 13.0 ± 7.4). There was no difference in time to resolution between patients who continued the culprit anticoagulant or changed to a different anticoagulant, and those who discontinued anticoagulation altogether (13.9 days vs. 12.1, p = 0.49). Four deaths have been reported in this clinical context, two specified as intracranial hemorrhage. These deaths were unrelated to the occurrence of BHD. Continuation of low-molecular weight heparins appeared to be safe in patients with BHD.

## Background

Low molecular weight heparins (LMWH) are extensively used as anticoagulation for primary and secondary prophylaxis against thrombosis, particularly in patients with malignancy. There are several known dermatological side-effects of LMWH, including injection site hematoma and skin necrosis, eczematous dermatitis, and the lesser known bullous hemorrhage dermatosis (BHD). The latter was first described in 2004 by Dyson et al. [1]. It typically presents as intra-epidermal lesions found mainly on the extremities and torso, with a black appearance. The lesions occur at sites distant from injection, suggesting a systemic mechanism. There have been several small case series on BHD, but none have captured a complete review of reported cases. The frequent use of LMWH warrants systematic review of this side-effect so clinicians recognize its characteristics and know how to intervene. We sought to describe a case of BHD at our institution and provide a complete summary of the existing English and Spanish literature.

## Methods

We performed a literature search of Pubmed, Scopus, Ovid Embase and Ovid Medline. The keywords for the search were “bullous” “hemorrhagic” and “dermatitis”. Of the initial 213 search results, 51 were found to be duplicates. The remaining 162 were reviewed by title and abstract. Of these, 33 were deemed relevant to BHD, and the entire article was reviewed. The majority of the excluded search results either did not include anticoagulant use or were relevant to dermatologic diseases other than BHD.

## Results

### Case presentation

**Fig. 1:**
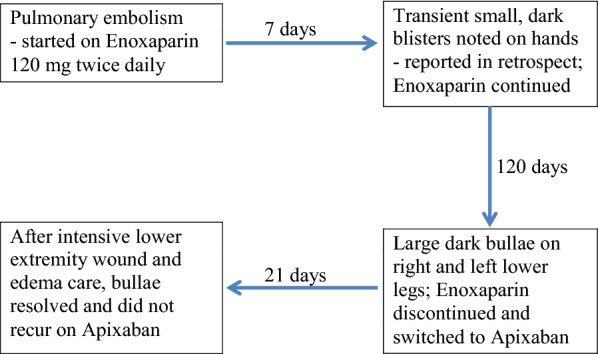
Timeline of clinical events

**Fig. 2:**
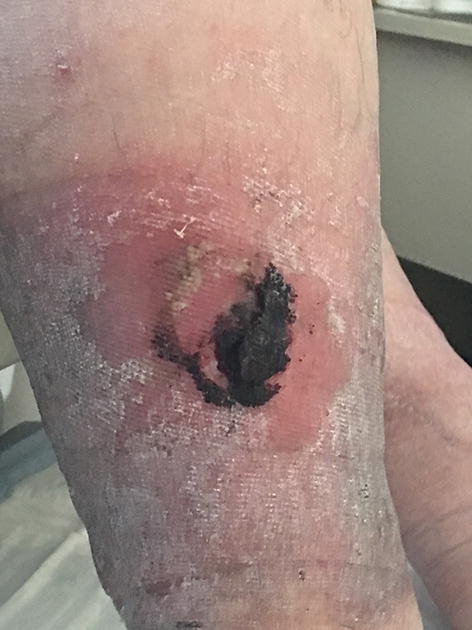
Left anterior lower extremity bulla

**Fig. 3:**
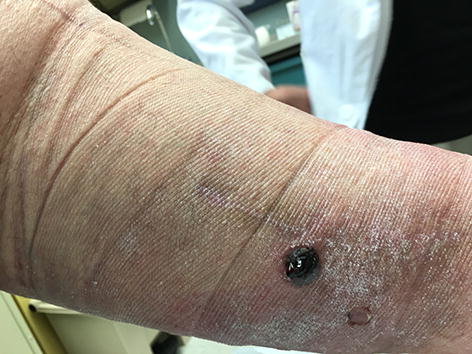
Right posterior lower extremity bulla

Our patient is a 62-year-old male with multiple comorbidities including morbid obesity, coronary artery disease, lipodermatosclerosis of the lower extremities, chronic peripheral venous insufficiency, and prostate cancer (Gleason 4+5) on long-term androgen deprivation therapy. He was previously treated with docetaxel for pelvic lymph node metastases. The patient also had a small renal tumor for which he was followed with imaging. He had a distant history of varicose vein ligation. While undergoing surveillance imaging to evaluate for spread of his prostate cancer, an incidental pulmonary embolism was discovered. He was started on enoxaparin 120 mg by subcutaneous injection twice daily. Within several days the patient noticed several small black blisters on his hands that resolved spontaneously. These were not reported by the patient or observed by any practitioner during routine clinic visits. The timeline of development of hemorrhagic skin lesions of this patient is outlined in Fig. 1. Four months after starting anticoagulation with enoxaparin, he presented with several large hemorrhagic bullae on his calves (Figs. 2 and 3). His coagulation values and his platelet count were within normal range. The diagnosis of bullous hemorrhagic dermatosis (BHD) was made by visual inspection. Therefore, no biopsy was performed. Enoxaparin was discontinued and apixaban was started as alternative anticoagulation. The lesions healed in 3 weeks with intensive outpatient wound care and have not recurred to date while on apixaban.

### Review of reported cases in the English and Spanish literature

We describe 90 cases of BHD discovered in our literature search, in addition to our local case (Table 1) [1–33]. Cases were reported from the US, Spain, France, Turkey, Austria, and Australia. Spain appeared to have the most representation in the literature. Serious comorbidities were common, such as carcinomas of lung, prostate, and ovary, lymphoma, and cardiomyopathy. The cases demonstrate a variety of indications and dosages for anticoagulation (not shown in table). Indications for weight-based, full dose anticoagulation included atrial fibrillation, venous thromboembolism, aortic stenosis, left ventricular thrombus, and acute coronary syndromes. Patients also received primary prophylactic dosing for hospitalized patients. Before their incident BHD, most patients had not been exposed to anticoagulation. A minority of patients received warfarin before diagnosis; none received direct oral anticoagulants (DOAC). Most patients had normal PT and PTT values before initiation of anticoagulation, and were in therapeutic range while on treatment (not shown). A minority of patients were on aspirin at the time of diagnosis. Most patients had a punch skin biopsy, and all were negative for signs of vasculitis. Intraepidermal hemorrhage was the common pathological finding. The eruption was mainly located on the extremities, and less frequently on the torso.

**Table 1:** Summary of cases (n = 91), part I, part II, part III

References	Age	Gender	Agent	Days to lesion	Location	Intervention	Days to resolution
Part I							
[1]	62	F	Enoxaparin	Unknown	Buttock	AC changed to warfarin	14
[1]	32	M	Enoxaparin	14	Lower extremities	AC discontinued	7
[2]	75	M	Dalteparin	5	Hands and groin	AC changed to coumadin, died of IC 14 days later	Death
[2]	82	F	Tinzaparin	6	Extremities	AC discontinued	10
[2]	64	M	Heparin Calcium	21	Forearms and ankles	AC continued	Yes, unknown
[3]	72	F	Enoxaparin	2	Abdomen and upper extremities	AC changed to oral after planned intervention	14
[3]	57	M	Enoxaparin	3	Abdomen and upper and lower extremities	Unknown	Unknown
[4]	88	M	Enoxaparin then warfarin	14	Left arm and ankle	AC discontinued	14
[5]	51	Unknown	Enoxaparin then tinzaparin	2	Upper and lower extremities, abdomen	AC discontinued	Unknown
[6]	86	M	Enoxaparin	1	Trunk, extremities	AC continued	30
[6]	87	M	Enoxaparin	5	Limbs and hands	AC continued	21
[6]	73	F	Bemiparin then Enoxaparin	1	Lower extremities	Changed to rivaroxaban, died 10 days later	Death
[6]	72	M	Bemiparin	30	Trunk, legs	AC continued	21
[6]	82	M	Enoxaparin	3	Arms	AC continued	21
[7]	68	M	Enoxaparin	8	Hand, neck, and face	Unknown	14
[7]	78	M	Enoxaparin	10	Knees and forearm	AC continued	14
[8]	85	M	Enoxaparin	3	Torso and legs	AC discontinued	Unknown
[9]	73	M	Enoxaparin	14	Upper extremities	AC continued	14
[10]	77	M	Enoxaparin	3	Trunk, right hand	AC discontinued	2
[10]	94	M	Tinzaparin	15	Trunk, limbs	AC discontinued	10
[11]	59	F	Enoxaparin	30	Dorsal hand	AC continued	4
[12]	68	M	Enoxaparin	1	Feet, leg, neck, hands dorsum	AC discontinued	Unknown
[12]	77	M	Enoxaparin	8	Dorsum of feet, dorsum of hands	AC discontinued	Unknown
[13]	88	F	Enoxaparin	5	Legs	AC changed to warfarin	Unknown
[14]	87	F	Enoxaparin	10	Knees, forearms, elbows	Unknown	14
[15]	71	M	Enoxaparin	2	Abdomen	AC discontinued	7
[16]	90	M	Enoxaparin	8	Left arm and ankle	AC continued	14
[17]	73	M	Enoxaparin	6	Left palm	AC continued	14
Part II							
[18]	63	M	Enoxaparin	12	Lower extremities	AC continued	3
[18]	74	M	Enoxaparin	13	Lower extremities	AC continued	3
[19]	83	M	Warfarin + UFH	5	Arms, flank, thighs	Heparin discontinued	Yes, unknown
[20]	77	M	Enoxaparin	5	Unknown	Changed to dabigatran	14
[21]	82	F	Warfarin	10	Extremities	Changed to enoxaparin	Worsening lesions, resolved after all AC stopped
[22]	90	M	Enoxaparin	8	Ankle and wrist	AC continued	14
[22]	65	M	Enoxaparin	9	Lower and upper extremities	Changed to tinzaparin as lesions persisted for 6 weeks on enoxaparin	14
[22]	64	M	Enoxaparin	7	Lower and upper extremities	AC continued	21
[22]	89	M	Enoxaparin	10	Lower and upper extremities scalp and back	AC discontinued	21
[22]	74	M	Enoxaparin	20	Hand and leg	AC discontinued	14
[23]	71	M	Enoxaparin	14	Forearms, hands, knees	AC continued	Persisting lesions for 6 months, resolved after AC stopped
[24]	64	M	Enoxaparin	5	Arms and hands	Dose reduced	14
[25]	74	M	Enoxaparin	5	Upper and lower extremities, abdomen	AC discontinued	7
[26]	52	M	Enoxaparin	7	Lower and upper extremities	AC continued	3
[27]	77	F	Enoxaparin	15	Limbs	AC continued	14
[28]	77	F	Enoxaparin	5	Hands and shins	AC continued, fatal ICH 4 weeks later	12
[29]	72	M	Enoxaparin	7	Arm and legs	AC changed to UFH	7
[30]	59	F	Enoxaparin	270	Legs	AC changed to fondaparinux then rivaroxaban	Unknown
[30]	51	F	Fondaparinux	Unknown	Legs	AC changed to rivaroxaban	3
[31]	75	F	Enoxaparin	3	Arms, hands, feet	AC held, lesions resolved, AC restarted and lesions re-appeared, improved with steroids, patient had past history of bullous pemphigoid	28
Part III							
[32]	60	M	UFH	5	Limbs and trunk	AC continued	Unknown
[32]	68	F	UFH then enoxaparin	7	Abdomen, hands, feet	AC changed to rivaroxaban	21
[32]	64	M	Enoxaparin, warfarin	9	Lower and upper extremities	AC discontinued	30
[32]	38	F	UFH	7	Chest and ankle	AC discontinued	7
[32]	72	F	Enoxaparin	2	Forearm	AC discontinued	2
Ref. [33]; Case series of 37 cases, 1985–2015	77.4	M 20 F 17	LMWH Fondaparinux Sodic heparin Calcic heparin	8.8	Limbs and trunk	AC discontinued in 34 cases AC continued in 1 case Unknown in 2 cases	Unknown
Case currently reported	62	M	Enoxaparin	7	Calf and hands	AC continued with worsening lesions, after 4 months changed to apixaban	21 after change to apixaban

*AC* anticoagulation, *F* female, *ICH* intracranial hemorrhage, *LMWH* low molecular weight heparin, *M* male, *UFH* unfractionated heparin

Summary statistics were extracted based on our review of the literature (Table 2). The average case age was 72 ± 12, and there was a 1.9:1 male to female ratio. Enoxaparin was the most frequent culprit anticoagulant (66%), followed by fondaparinux (12%). On average, lesions appeared after 7 days of treatment (7.0 ± 6.4), and resolved after 2 weeks of discontinuation of anticoagulation (13.0 ± 7.4). Most cases of bullous lesions (67.9%) were reported on the extremities only, followed by torso and extremities (26.4% of cases). Lesions on the head and neck were rare (5.5%). Management consisted of discontinuation of culprit anticoagulant (57% of cases), continuation (23.1%), or treatment change or dose reduction (14.3%). There was no difference in time to resolution between patients who continued the culprit anticoagulant or changed to a different anticoagulant, and those who discontinued anticoagulation altogether (13.9 days vs. 12.1, p = 0.49).

**Table 2:** Descriptive statistics from 91 cases of BHD

Descriptor	Statistic
Age in years, average ± SD	72 ± 12
Sex % (n)	
Male	64% (58)
Female	36% (33)
Anticoagulation drug, % (n)	
Enoxaparin only	66% (61)
Fondaparinux	12% (11)
UFH only	9% (8)
Bridging heparin + enoxaparin	6.5% (6)
Other LMWH or ULMWH	5.4% (5)
Coumadin only	1% (1)
Time to lesion onset, days, mean ± SD	7.0 ± 6.4
Location % (n)	
Extremities only	67.9% (36)
Extremities +torso	26.4% (14)
Face, neck, head included	5.7% (3)
Management % (n)	
Discontinued	57% (52)
Continued	23.1% (21)
Treatment changed or dose reduced	14.3% (13)
Unknown	5.5% (5)
Time to resolution, days, mean ± SD	13.0 ± 7.4
If continued any AC	13.9 ± 7.8
Discontinued AC	12.1 ± 7.9

## Discussion

The review of BHD presented here is the largest collection of cases compiled from the English and Spanish literature to our knowledge. BHD has been documented in medical reports, but appears under-recognized in the clinical practice. We also presented a case from our own institution.

BHD is a non-immune dermatological eruption related to anticoagulation given for either primary or secondary prophylaxis. Enoxaparin is the most common culprit anticoagulant, followed by fondaparinux. Lesions are distant from the site of injection. Clinical or pathological signs of inflammation are absent. The extremities are the primary sites of eruptions in many cases. BHD caused by DOACs is not specified to date from several examples of large clinical trials for VTE treatment [34–36]. No patients in our series received DOACs before diagnosis.

Our case is unusual for the delay of 4 months in the appearance of BHD consisting of very large lesions in the lower extremities. Most lesions will appear within 7 days of the first anticoagulation administration. Their appearance is usually of small, dark bullae, rather than large hemorrhagic bullae. Lesions are not painful. In retrospect, our patient did recall having small blisters on his hands that occurred within days of starting enoxaparin. However, he did not report these small skin lesions as they resolved spontaneously. He sought medical attention after developing large dark bullae on both calves.

From our viewpoint, if characteristics of BHD are present as outlined above and in Table 2, historical and clinical evaluation may be satisfactory to make the diagnosis. A biopsy should be performed if the diagnosis is in doubt. In our patient’s case, prostate cancer treatment with androgen deprivation is an unlikely cause. While injection-site granuloma and serum sickness have been reported with depot leuprolide acetate [37–39], to our knowledge, there are no reports of eruptions similar to BHD with leuprolide.

After the appearance of BHD, maintaining anticoagulation with a LMWH may increase a patient’s risk of recurrent bullae [21, 22, 31]. Nevertheless, 21 patients in our review continued on anticoagulation with a LMWH without report of recurrence. It is suspected that higher doses of LMWH may increase time to resolution of BHD [22]. No treatment is needed beyond managing anticoagulation and skin care as in this case. Discontinuing treatment may not be necessary if lesions are small.

BHD is sometimes associated with eczematous reaction at sight of injection that may suggest a type IV hypersensitivity reaction [32]. The propensity for developing lesions on the extremities in older patients also posits local trauma combined with epidermal-dermal fragility as an underlying mechanism [10]. There is no apparent association of BHD with any comorbidity. While there is no evidence indicating a higher risk for BHD with venous insufficiency, one could speculate that the pre-existing chronic leg edema in our patient facilitated the large size of the bullous lesions.

Of the 91 cases presented, there were four cases that had bleeding complications, two with fatal intracranial hemorrhage [2, 28]. Available coagulation tests were normal in these patients at the onset of anticoagulation. In Perrinaud et al. [2], the bleeding event occurred with a supratherapeutic INR. In Yurekli et al. [28], the fatal event occurred after continuing enoxaparin, but the bullous lesions had resolved at the time of death. The documented rate of intracranial hemorrhage in the CLOT trial while using LMWH for a 6-month study period was one out of 338 [40].

## Conclusion

Hemorrhagic bullous dermatosis secondary to LMWH usage has been reported in the literature but is under-recognized. In this case report the diagnosis was not made until the patient presented with large hemorrhagic bullae after 4 months of LMWH. Biopsy may not be required if the clinical characteristics of BHD are present. The anticoagulation of choice may be continued if lesions are small and are resolving. Risk evaluation for bleeding may proceed along standard guidelines. This case series also highlights a need for clinical reporting of BHD when using DOACs.
